# Characteristics and Outcomes of Chinese Children With Advanced Stage Anaplastic Large Cell Lymphoma: A Single-Center Experience

**DOI:** 10.3389/fonc.2022.832752

**Published:** 2022-02-15

**Authors:** Yu-Tong Zhang, Li-Zhe Wang, Jian Chang

**Affiliations:** Department of Pediatric Oncology, The First Hospital of Jilin University, Changchun, China

**Keywords:** risk factors, LDH, APO regimen, crizotinib, relapsed, ALCL, pediatric

## Abstract

**Purpose:**

To evaluate the clinical characteristics and treatment outcomes of Chinese children with advanced stage anaplastic large cell lymphoma (ALCL) who were treated with the low-intensity APO regimen.

**Methods:**

Clinical data from children newly diagnosed with advanced stage ALCL and treated with the APO regimen were reviewed.

**Results:**

Altogether 22 eligible patients with advanced stage ALCL were recruited in this study. 18 (81%) patients achieved complete response (CR) after the initial induction, and 4 experienced relapse. Among patients with relapsed or refractory ALCL, CR was achieved in 3 (50%) who received the BFM95 R3/R4 regimen. Besides, 2 patients received the targeted therapy with crizotinib and were still alive. The 5-year OS and EFS rates were 82 ± 8.7% and 68.2 ± 9.4%%, respectively. According to our results, the elevated LDH level and bone marrow involvement were identified as the poor prognostic factors for EFS (p=0.035 and 0.048, respectively). During APO treatment, only 23% patients experienced grade 3-4 hematologic toxicity.

**Conclusions:**

In this study, bone marrow involvement and elevated serum LDH levels were identified as the poor prognostic factors for EFS. In resource-limited regions, patients with advanced stage ALCL can also achieve comparable outcomes to those in high-income regions, and the BFM95 R3/R4 regimen can take the role of salvage treatment for patients with relapsed or refractory disease. Nonetheless, new therapeutic strategy is still needed.

## Introduction

Anaplastic large cell lymphoma (ALCL) is a rare disease in children, which accounts for 10%-15% of all childhood non-Hodgkin’s lymphomas (1). According to the latest WHO classification of lymphoma, ALCL is restricted to lymphoma with a T/NK-cell or null-cell phenotype, and is strongly positive for CD30 (2). Over the past two decades, ALCL has been increasingly characterized, and is becoming one of the most curable childhood malignancies in high-income countries. However, the optimal treatment for pediatric advanced stage ALCL has not been established in terms of its efficacy and safety, especially for patients with relapsed or refractory disease. At present, pediatric ALCL is mostly understood from European and American studies, but rarely from Asian studies. Besides, most of the existing therapeutic strategies mainly investigate the intensive short-pulse chemotherapy regimen based on B-cell NHL-type therapy (3, 4) and prolonged repeated-pulse APO (doxorubicin, predisone, vincristine, methotrexate, 6-mercaptopurine) regimen (5, 6). Despite the variations in regimens, the event-free survival (EFS) in ALCL children is highly consistent across different pediatric studies, with a 2-year failure rate of approximately 20-30% (4–6). In this regard, it is necessary to consider the compliance and availability of a therapeutic regimen, especially in resource-limited regions. Therefore, this study was designed to evaluate the clinical characteristics and outcomes of Chinese children with advanced stage ALCL who were treated with the low-intensity APO regimen at our institute.

## Patients and Methods

### Patients and Diagnosis

The medical records of patients aged ≤18 years who were diagnosed with advanced stage ALCL at our institute were reviewed from January 2007 to January 2016. This retrospective study was approved by the Institute Ethical Committee. Due to the retrospective nature of the study, the requirement for written informed consent was waived. The diagnoses in patients were confirmed according to the histopathological and immunohistochemical criteria defined by the 2008 WHO classification. Moreover, all of the radiographic and pathological data were reviewed to confirm the diagnosis and staging by one independent radiologist and pathologist, respectively,.

### Staging

All patients were staged in line with the St Jude staging system (7). At diagnosis, the staging work-up included a complete blood cell count, serum biochemistry, serum levels of lactate dehydrogenase (LDH) and ferritin, cerebrospinal fluid (CSF) analysis, bone marrow examination, pleural fluid and ascites analyses, computed tomography (CT), magnetic resonance imaging (MRI) or PET scan to determine the tumor extent.

Advanced stage ALCL was defined as stage III or IV disease according to the St Jude staging system or disease with multiple involvements of bone, mediastinum, viscera (lung, liver and spleen), skin or soft tissue.

### Treatment

Patients with advanced stage ALCL were all treated by adopting the APO regimen based on the standard arm of the Pediatric Oncology Group protocol POG-9315 (5). Specifically, induction therapy consisted of doxorubicin at 75 mg/m^2^ on days 1 and 22; vincristine at 1.5 mg/m^2^ on days 1, 8, 15, 22 and 29; prednisone at 40 mg/m^2^ daily for 28 days; and age-adjusted intrathecal methotrexate on days 1, 8, and 22. Besides, the maintenance therapy included 15 cycles of doxorubicin at 30 mg/m^2^ on day1 (cycles 1-5), methotrexate at 60 mg/m^2^ on day1 (cycles 6-15), vincristine at 1.5 mg/m^2^ on day1, prednisone at 120 mg/m^2^ on days 1-5, and 6-mercaptopurine at 225/mg/m^2^ on days 1-5, given at intervals of 21 days for an approximate duration of 12 months.

For patients who developed relapse or progressive disease (PD), or those who did not achieve complete response (CR) after induction therapy, vinblastine or targeted therapy with crizotinib or stem cell transplantation (SCT) was recommended. However, for patients who could not reach this stage, the salvage treatment was taken with the BFM-95 protocol R3/R4 arm (AA → BB →CC→AA→BB → CC) (8).

### Treatment Response

CR was defined as the disappearance of disease or residual lesion with a negative PET scan. Partial response (PR) was defined as >50% tumor regression in the perpendicular diameters of the lesions, with no new lesion formation. Treatment response was assessed at the end of induction therapy. Non-response (NR) was assumed in the presence of ≤30% tumor regression. Stable disease (SD) was defined as neither PR nor PD criteria met. PD was defined as the appearance of new lesion during treatment or evidence of primary tumor progression. Relapse was defined as evidence of disease at 1 month after the completion of chemotherapy.

### Statistics

Overall survival (OS) was defined as the duration from diagnosis to death or the last follow-up. EFS was defined as the duration from diagnosis to the occurrence of the first event, including disease progression, disease recurrence or death. The OS and EFS rates were estimated using the Kaplan-Meier method. Meanwhile, difference in survival was assessed by a log-rank test. The Cox proportional hazards model was employed to analyze the impact of potential prognostic factors on OS and EFS. Statistical analyses were performed using the software R version2.9.1 for Windows (www.r-project.org).

## Results

### Clinical Characteristics

Altogether 22 eligible patients with advanced ALCL were recruited into this study, with a median age of 12.5 (range, 9-18) years at diagnosis. The demographic and clinical characteristics of these 22 patients are summarized in **Table 1**. There were 10 male (45.5%) and 12 female (54.5%) patients. According to the St. Jude staging system, 16 (72.7%) patients were at stage III, while 6 (27.3%) were at stage IV. At the time of diagnosis, 86.4% of patients were detected with nodal involvement and 77.3% with extranodal involvement (including 53% with multiple extranodal involvements). In our cohort, the most common site of extranodal involvement was mediastinum, followed by bone (27.3%), bone marrow (22.7%), and lung or liver (18.2%). Meanwhile, as discovered from the biochemical records, 16 (72.7%) patients had elevated serum ferritin levels, and 19 (86.3%) had normal serum LDH levels <500 U/L. The ALK status was available in 21 patients, and 19 of them were positive.

**Table 1:** Demographic and clinical characteristics of 21 children with advanced stage ALCL.

Characteristics		N (%)
Age at diagnosis (years)	Median age 11.1 (7.2-15)	
	<10 year	3 (13.6%)
	≥10 year	19 (86.4%)
Gender	Male	10 (45.5%)
	Female	12 (54.5%)
ALK status	Positive	19 (86.4%)
	Negative	2 (9.1%)
	Unknown	1 (5.5%)
Stage	III	16 (72.7%)
	IV	6 (27.3%)
Lymph node involvement		19 (86.4%)
Extranodal involvement		17 (77.3%)
	Skin/soft tissue	3 (13.6%)
	Mediastinum	10 (45.5%)
	CNS	3 (13.6%)
	Liver	4 (18.2%)
	Spleen	2 (9.1%)
	Lung	4 (18.2%)
	Other visceral involvement	2 (9.1%)
	Bone	5 (22.7%)
	Bone marrow	6 (27.3%)
B symptoms	Absent	14 (63.6%)
	Present	8 (36.4%)
Serum LDH level	≤500 IU/L	19 (86.3%)
	>500 IU/L	3 (13.6%)
Serum Ferritin level	≤150 ug/L	6 (27.3%)
	>150 ug/L	16 (72.7%)
Morphologic subtype	Common	17 (77.3%)
	Small cell	2 (9.1%)
	Other type	3 (13.6%)
Events	Relapse	4 (18.2%)
	Progressive disease	3 (13.6%)


CNS, central nervous system; ALK, anaplastic lymphoma kinase; LDH, lactate dehydrogenase.

### Treatment Outcomes

**Figure 1:**
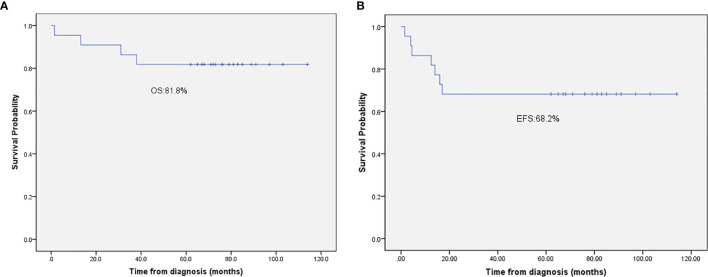
**(A)** The 5-year overall survival rate of patients with advanced-stage ALCL. **(B)** The 5-year event-free survival rate of patients with advanced-stage ALCL.

During the induction therapy, 1 patient (No. 18) with CNS involvement died of respiratory failure. For the remaining 21 patients, 18 (81%) patients achieved CR and 3 (13.5%) attained PR after initial induction therapy. The 3 patients with PR received salvage treatment with the BFM95 R3/R4 regimen. Among them, one patient (No.7) with ALK-negative ALCL still presented with refractory disease after treatment with the BFM95 R3/R4 regimen, therefore gave up on the treatment; the other 2 patients reached CR after 3 cycles of chemotherapy, but both of them experienced progressive disease in skin (No.6) or relapse in lymph nodes (No.4), respectively. They both received crizotinib (an oral target drug), and are still alive. For patients who achieved CR after initial induction therapy, 3 patients had relapsed during follow-up, so all of them received salvage treatment with the BFM95 R3/R4 regimen. 1 patient (No.12) died of disease progression. 2 patients achieved second CR, 1 (No.9) continued to be CR, while the other one (No.19) with ALK-negative ALCL received allogeneic SCT, but died of disease 1 year after bone marrow transplantation. After a median follow-up period of 65 (rang, 13.2-114) months, the 5-year OS and EFS rates were 82% (95%CI of 72.6-91.4%) and 68.2% (95%CI of 59.5-76.9%), respectively (**Figure 1**). The cumulative incidence of relapse and PD in this study was 31%.

In total, through salvage chemotherapy with the BFM95 R3/R4 regimen, CR was achieved in 3 (50%) patients with refractory or relapsed disease. Meanwhile, the 3 patients who had elevated serum LDH levels at diagnosis suffered relapse or refractory disease and all died of this disease; and two of whom were confirmed with ALK-negative ALCL. Additional details are displayed in **Table 2**.

**Table 2:** Characteristics and outcomes of the patients with relapsed or refractory ALCL.

No.	Stage at initial diagnosis	Gender/Age(years)	ALK status	Serum LDH level at diagnosis (U/L)	Serum ferritin level at diagnosis (ug/L)	Response to initial induction	Pattern of event (R/PD/Sites)	Time of events from diagnosis (months)	Salvage therapy	Status
4	IV	F/10	+	220	129	PR	R/LN	21	BFM95/Crizotinib	Alive
9	IV	M/12	+	88	225	CR	R/LN/S	18.0	BFM95	Alive
12	IV	M/13	+	390	243	CR	R/LN/BM	14	BFM95	Death
19	IV	F/16	–	845	542	CR	R/LN/	16.0	BFM95/SCT	Death
6	IV	F/13	+	360	504	PR	PD/S	4.0	BFM95/Crizotinib	Alive
7	III	M/18	–	1597	183	PR	PD	4.5	BFM95 (NR to AA+BB+CC)	Death
16	IV	M/12	+	1293	21450	NR	PD	1.5	–	Death

M, male; F, female; LDH, lactic dehydrogenase; R, relapse; PD, progressive disease; ALCL, anaplastic large cell lymphoma; LN, lymph node; S, skin; BM, bone marrow; CR, complete response; PR, partial response; NR, no response; SCT, stem cell transplantation.

### Treatment Toxicity

For all patients, grade 3-4 myelosuppression was the main treatment-related toxicity during the front-line therapy, which was under control. In addition, grade 4 hematologic toxicity occurred in 23% patients, which lasted for less than 2 weeks. The other toxicities included mild liver dysfunction, nausea, and vomiting.

### Prognostic Factors

As revealed by log-rank analysis, stage, bone marrow involvement and elevated LDH level were the significant influencing factors for EFS (**Table 3**). There was a tendency that most patients with refractory and relapsed ALCL had elevated ferritin levels, but the difference was not significant (5-year EFS, 87.9% *vs* 60,2%, *p*=0.29). In multivariate Cox regression analysis, only bone marrow involvement and elevated serum LDH level were identified as the significant adverse prognostic factors for EFS (*p*=0.048, and 0.035, respectively).

**Table 3:** Prognostic factors for overall survival and event-free survival rates (log-rank test).

Factors		5-year EFS rate	*P*	5-year OS rate	*P*
Age at diagnosis(year)	<10 year	100%	0.248	100%	0.407
	≥10 year	63.2%		78.9%	
Gender	Male	60.1%	0.468	70.0%	0.190
	Female	75.0%		91.7%	
Stage	III	81.6.2%	0.045	89.7%	0.180
	IV	45.4%		57.1%	
Lymph node involvement Yes		66.7%	0.530	81.0%	0.650
No		100%		100%	
Extranodal involvement	Yes	58.8%	0.110	76.5%	0.255
	No	100%		100%	
Skin and soft tissue	Yes	33.3%	0.176	100%	0.407
	No	73.7%		78.9%	
CNS	Yes	33.3%	0.148	66.7%	0.366
	No	73.7%		84.2%	
Mediastimum	Yes	60.0%	0.433	80.0%	0.842
	No	75.0%		83.3%	
Lung	Yes	69.6%	0.896	76.0%	0.218
	No	87.5%		93.0%	
Bone marrow	Yes	16.7%	0.002	66.7%	0.382
	No	87.5%		87.5%	
Bone	Yes	80.0%	0.460	100%	0.255
	No	64.7%		76.5%	
B symptoms	Yes	69.5%	0.49	75.8%	0.56
	No	85.4%		89.1%	
Visceral involvement	Yes	60.4%	0.966	66.7%	0.328
	No	68.8%		87.5%	
Serum LDH level	≤500 U/L	0.789%	0.001	94.7%	0.001
	>500 U/L	0.00%		0.00%	
Serum Ferritin level	≤150ug/L	83.2%	0.293	100%	0.164
	>150ug/L	57.1%		71.4%	

CNS, central nervous system; ALK, anaplastic lymphoma kinase; LDH, lactate dehydrogenase.

## Discussion

As a relative rare subtype of childhood lymphoma, ALCL displays unique characteristics. More than 90% of ALCL children are positive for ALK, which is mainly ascribed to the translocation t(2:5)(p23;q35) involving the ALK and NPM genes (9). Most pediatric patients with ALCL present with advanced stage disease, and almost all of them have nodal involvement, and extranodal involvement is also common (3, 4). To date, there are only 4 clinical reports available on pediatric ALCL from East Asia (10–13). To confirm the optimal therapy for pediatric ALCL in the resource-limited regions, this study reviewed 22 Chinese pediatric patients diagnosed with advanced stage ALCL, to establish clinical features, outcomes, prognostic factors, and treatments of refractory or relapsed disease in China. In this study, the clinical characteristics of patients with advanced stage ALCL were similar to those reported in other Asian studies (10–13). Typically, bone marrow involvement and elevated serum LDH levels were identified as the adverse prognostic factors for EFS. Combined with other reports from East Asia, bone marrow involvement appears to be a consistent adverse prognostic factor for EFS in East Asian children with ALCL (10, 11). Of note, most patients (92%) in this cohort presented with normal LDH levels at diagnosis, which were not associated with disease stage, PD, or relapse, but the 3 patients with high LDH levels at diagnosis died of this disease. In a small cohort study on 39 Chinese children with ALCL, Sun *et al.* also detected a similar characteristic (12). Therefore, it seems that the elevated LDH levels at diagnosis is not an indicator of disease extent or metastases in ALCL, rather than in other pediatric lymphoma subtypes (14), and it is predictive of the terminal phase of disease. So far, no clinical features have been consistently identified as the prognostic factors for survival in ALCL children (10, 15, 16). This may be explained by the following reasons: ①most clinical results come from a small cohort study; ②the enrollment criteria are varies greatly between studies; ③ there is no common definition, such as mediastinal involvement and liver/spleen involvement; and ④the initial therapy and salvage treatment strategies are different. In a large-scale European intergroup study enrolling 225 patients, skin lesions, B symptoms, mediastinal involvement, visceral involvement and elevated LDH level were identified as the adverse prognostic factors (17). However, in a recent report on the largest cohort of 420 ALCL patients from the European Inter-Group for Childhood Non-Hodgkin lymphoma (EICNHL), only the small cell/lymphohistocytic pattern was independently associated with treatment failure among the clinical/pathological characteristics in children (16). Overall, definitive prognostic factors for survival can contribute to formulating the risk stratification therapy, understanding the biological/pathological characterizations of pediatric patients with poor outcomes, and designing future therapeutic strategies.

One of the characteristics of childhood ALCL is its chemosensitivity in front-line therapy and at relapse, which shows a high response rate to various chemotherapy regimens and achieves a very similar EFS rate of about 65-80% (3, 6). Given low cumulative doses of anthracyclines and alkylating agents, which are associated with the long-term toxicities, most pediatric groups have adopted the NHL-BFM90 regimen-based ALCL99 chemotherapy. However, it is related to acute toxicities, among which, grade IV neutropenia is reported in 60% patients, and mucositis is detected in 15% cases (18). Compared with the low-intensity prolonged APO regimen, the acute toxicity of ALCL99 regimen reduces the patient compliance to some extent and increases the requirement of inpatient care in resource-limited regions. Therefore, at our institute, the APO regimen, which was a 12-month outpatient regimen that avoided the use of alkylating agents, was taken as the front-line therapy for patients with advanced stage ALCL. In this study, 81% patients achieved CR after the initial induction therapy, grade 4 hematologic toxicity occurred in 23% patients for less than 2 weeks, and no patient was recorded with anthracyclines -related toxicity. After a median follow-up period of 65 (range, 13.2-114) months, the 5-year OS and EFS rates were 82% and 68.2%, respectively, which were comparable to those previously reported (10–12). In a small cohort on 31 children with ALCL, Ceppi *et al.* reported that treatment-related mortality was the major cause of treatment failure for children with ALCL in low- and middle-income countries (19). Therefore, chemotherapy may be a crucial option to ensure the patient compliance and reduce treatment-related failure in resource-limited regions.

In total, 7 patients suffered PD or relapsed disease in this study, and no standard established treatment was recommended for these patients. However, based on the 5-year OS rate of 82%, it is suggested that patients with refractory or relapsed disease may survive after the current salvage treatments. The recommendations of salvage treatment vary among different pediatric groups; particularly, the option is largely limited in resource-limited regions. In this study, 6 patients suffering from refractory or relapsed disease received the salvage treatment with the BFM95 R3/R4 regimen at first; among them, CR was achieved in 3 (50%) patients. Case 9 (a relapsed patient) remained disease free for 76 months after the BFM95 R3/R4 therapy alone. This suggests that salvage treatment with the intensive BFM95 R3/R4 regimen may be an option for patients with refractory or relapsed ALCL in resource-limited regions. As confirmed by results obtained from the prospective clinical trial launched by EICNHL, allogenetic SCT achieved good efficacy in high-risk relapsed patients (20). However, due to the economic reasons, allogenetic SCT is not available for every relapsed patient in resource-limited regions. In this study, only one patient with ALK-negative ALCL received SCT, and still died of disease progression.

The activity of ALK tyrosine kinase is essential to the survival of ALK+ ALCL, in this regard, the ALK inhibitors show promising prospects in the management of ALK+ ALCL patients. As shown by some small cohort studies, crizotinib can induce CR in most relapsed patients (21), with no disease progression in the course of crizotinib treatment (22). However, it has been confirmed that crizotinib is not curative, and abrupt relapse is frequent after crizotinib discontinuation (23). So far, the optimal duration and administration mode of crizotinib have not been defined yet. At present, a clinical trial conducted by the COG group is ongoing to test the efficacy of crizotinib in ALCL children (NCT01979536). Besides, the EICNHL group is also evaluating the efficacy and safety of crizotinib in combination with chemotherapy in ALCL children. In our study, Case 4 and Case 6 with second disease progression received crizotinib and remain alive now. Of the two, Case 6 received crizotinib in combination with BFM95 R3/R4 therapy, due to the formation of new skin lesion in the course of BFM95 chemotherapy, and no severe toxicity was recorded. We expect that results from the EICNHL group test the advantage of the combination therapy involving crizotinib and conventional chemotherapy.

In addition to ALK inhibitor agents, vinblastine is another promising novel agent that is being investigated in various ongoing strategies for ALCL, since the first report demonstrating the high efficacy of vinblastine in patients with relapsed ALCL (24). However, vinblastine is not available in China, so none of the patients in the study received this drug as salvage treatment. Additionally, a randomized trial showed that APO (ANHL0131) chemotherapy in combination with weekly vinblastine in maintenance phase has no effect on improving EFS or OS (5). Furthermore, the results obtained from ALCL-99 also indicate that the length of therapy, rather than the addition of vinblastine, results in delayed relapse (4). Therefore, children with ALCL may benefit from the treatment strategy established based on the prolonged APO regimen. Overall, new therapeutic agents and targeted therapies, such as vinblastine, crizotinib and brentuximab vedotin, have recently exhibited promising results (25), but their application in resource-limited regions is largely limited by their availability and costs. In the future, the availability of these new therapeutic options will certainly spare low-risk patients from acute toxicity and reduce the treatment failure rate in high-risk patients.

According to the results from our small cohort study, patients with advanced ALCL in resource-limited regions can also achieve comparable outcomes to those in high-income regions. The future treatment strategy for childhood ALCL should be designed based on the low-intensity prolonged APO therapy, which can delay the occurrence of relapse. Moreover, more caution should be paid to patients with elevated LDH levels at diagnosis, and conventional chemotherapy in combination with targeted therapy may be an option for advanced stage ALCL patients. We hope that our experiences can be tested in the future large-scale clinical trials.

## Data Availability Statement

The original contributions presented in the study are included in the article/supplementary material. Further inquiries can be directed to the corresponding author.

## Ethics Statement

The studies involving human participants were reviewed and approved by First Hospital of Jilin University. Written informed consent to participate in this study was provided by the participants’ legal guardian/next of kin.

## Author Contributions

Y-TZ, L-ZW, and JC made substantial contributions to design of the work and drafted the manuscript. All authors have read and approved the manuscript and agree to be accountable for all aspects of the work in ensuring that questions related to the accuracy or integrity of any part of the work are appropriately investigated and resolved.

## Conflict of Interest

The authors declare that the research was conducted in the absence of any commercial or financial relationships that could be construed as a potential conflict of interest.

## Publisher’s Note

All claims expressed in this article are solely those of the authors and do not necessarily represent those of their affiliated organizations, or those of the publisher, the editors and the reviewers. Any product that may be evaluated in this article, or claim that may be made by its manufacturer, is not guaranteed or endorsed by the publisher.

## Abbreviations

ALCL, anaplastic large cell lymphoma; ALCL99, anaplastic large cell lymphoma99 therapy; APO, APO (doxorubicin, predisone, vincristine, methotrexate, 6-mercaptopurine) regimen; CNS, central nervous system; CR, complete response; CSF, cerebrospinal fluid; CT, computed tomography; EFS, event-free survival; EICNHL, European Inter-Group for Childhood Non-Hodgkin lymphoma; LDH, lactate dehydrogenase; MRI, magnetic resonance imaging; NR, Non-response; OS, Overall survival; PD, progressive disease; PR, Partial response; R3/R4 therapy, BFM-95 protocol R3/R4 arm (AA → BB →CC→AA→BB → CC); SCT, stem cell transplantation.
